# Phase-Modulated Standing Wave Interferometer

**DOI:** 10.3390/mi12040357

**Published:** 2021-03-25

**Authors:** Ingo Ortlepp, Eberhard Manske, Jens-Peter Zöllner, Ivo W. Rangelow

**Affiliations:** 1 Institute of Process Measurement and Sensor Technology, Technische Universität Ilmenau, 98693 Ilmenau, Germany; eberhard.manske@tu-ilmenau.de; 2 Group: Microelectronic and Nanoelectronic Systems, Technische Universität Ilmenau, 98693 Ilmenau, Germany; jens-peter.zoellner@tu-ilmenau.de; 3 Nanoscale Systems Group, Technische Universität Ilmenau, 98693 Ilmenau, Germany; ivo.rangelow@tu-ilmenau.de

**Keywords:** standing wave, interferometer, photo sensor, phase modulation

## Abstract

The actual technical implementation of conventional interferometers is quite complex and requires manual manufacturing. In combination with the required construction space defined by the optical setup, their applications are limited to selected measuring tasks. In contrast, Standing Wave Interferometers (*SWI*s) offer an enormous potential for miniaturisation because of their simple linear optical setup, consisting only of a laser source, a measuring mirror and two transparent standing wave sensors for obtaining quadrature signals. The two sensors are located inside the measuring beam and therefore directly influence the length measurement. To reduce optical influences on the standing wave and avoid the need for an exact and long-term stable sensor-to-sensor-distance, a single sensor configuration was developed. There, a phase modulation is superimposed to the sensor signal by a forced oscillation of the measuring mirror. When the correct modulation stroke is applied, the resulting harmonics in the sensor signal are 90° phase-shifted to each other and can hence be used for obtaining quadrature signals for phase demodulation and direction discrimination by an arctan-algorithm.

## 1. Introduction

For measurement tasks with the highest requirements on resolution and measurement uncertainty, for calibrations, in semiconductor manufacturing and other precision technologies, interferometric measurement methods have been beneficially used for decades and are the means of choice today. A large portion of the measurement tasks are length measurements, where the Michelson/interferometer is commonly used in one of its many variants. In contrast to the simple basic principle of most interferometer types, their real technical implementation is often extremely complex. The multitude of optical components requires an enormous effort during manufacturing, assembly and adjustment. For this reason, commercially available interferometers are price-intensive and still represent a niche product for high technology. Furthermore, because of the cross-wise optical beam path, the miniaturisation of such interferometers is limited.

In this paper, an interferometric concept is proposed, which can overcome these limitations. It is based on an optical standing wave, which arises when a laser beam hits a mirror and is reflected into itself, and a single transparent photo detector.

With this concept, the assembly and adjustment effort as well as the space requirements can be significantly reduced compared to a conventional Michelson/interferometer. The *SWI* offers a simple linear structure with only a small amount of components [1,2]. It consists only of a laser source, a thin, transparent photo sensor and a modulated measuring mirror.

The phase-modulated *SWI* described in this manuscript utilises an additional modulation of the measuring mirror, superimposing the movement induced by the measurement. This way, the requirements for the transparent photo sensors can be significantly reduced, which are quite tight for a homodyne *SWI* [3]. There, two stable 90°-phase shifted interference signals are necessary, which results in a tolerance for the sensor distance in the nanometre range. With the proposed structure, a phase-modulated *SWI* offers an excellent potential for miniaturising the complete interferometer down to a single transparent photo sensor with dimensions only slightly larger than the beam diameter. Additionally, these sensors can be manufactured with standard semiconductor technologies, enabling a cheap mass production of interferometer components.

## 2. State of the Art

### 2.1. The Optical Stranding Wave

The interference of electromagnetic waves is typically considered as the interaction of two waves of the same wavelength, propagating in the same direction, resulting in a static interference pattern [4]. In contrast, in the *SWI*, the interference occurs between two waves propagating and interfering in opposite directions. This results in an optical standing wave, which was first demonstrated by Wiener [5].

The electric field of plane and monochromatic wave with a wavelength λ can be described by

(1) $$E\left(z,t\right)=E\cos\left(\frac{2\pi }{\lambda }\left(z-ct\right)\right)\;,$$

with the optical axis *z*, phase velocity *c*, time *t* and the amplitude E.

In the homodyne case of an *SWI*, a counter-propagating wave is created by inserting a mirror in the optical path at the position z_m_. Then, the electric fields of incident wave $E_{i}$ and reflected wave $E_{r}$ are

(2) $$\begin{array}{cc} E_{i} & =E_{i}\cos\left(\frac{2\pi }{\lambda }\left(z-ct\right)\right)\;\mathrm{and} \end{array}$$

(3) $$\begin{array}{cc} E_{r} & =E_{r}\cos\left(\frac{2\pi }{\lambda }\left(2z_{m}-z-ct\right)+\pi \right). \end{array}$$

There, π is the phase shift, occurring when the incident wave is reflected at the mirror.

Incident and reflected wave interfere in opposite directions. The resulting electric field $E_{\mathrm{SW}}$ can be calculated by the superposition of $E_{i}$ and $E_{r}$. For an ideal mirror, the amplitudes of incident and reflected wave are equal, resulting in

(4) $$E_{\mathrm{SW}}=2E_{i}\sin\left(\frac{2\pi }{\lambda }ct\right)\;\cdot \;\sin\left(\frac{2\pi }{\lambda }\left(z-z_{m}\right)\right)\;.$$

As can be deduced from Equation (4), the electric field of the standing wave consists of a time dependent and a position dependent term, where periodic zero points exist at $n\;\frac{\lambda }{2}$, n∈Z. Furthermore, there are periodic nodes and antinodes along the optical axis. Thus, the resulting interference pattern forms a stationary standing wave. It can be shown that the electric field profile is coupled to the mirror surface [6] so that moving the mirror along the optical axis will also shift the periodic profile. These properties of the electric field $E_{\mathrm{SW}}$ allow for a length measurement by evaluating the position of the nodes and antinodes. However, photo detectors do not detect the electrical field directly, but the time average of the Poynting-vector, the intensity of the wave. The intensity $I_{\mathrm{SW}}$ of the electric field given in Equation (4) is

(5) $$I_{\mathrm{SW}}=4I_{i}\sin^{2}\left(\frac{2\pi }{\lambda }\left(z-z_{m}\right)\right)\;.$$

**Figure 1:**
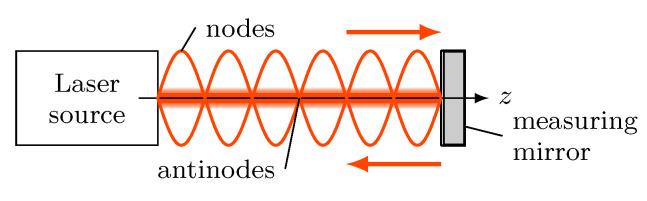
Stationary homodyne standing wave between a laser source and a measuring mirror. The intensity profile of the standing wave is phase coupled to the mirror surface, so moving the mirror will also shift the standing wave along the optical axis z.

The intensity profile $I_{\mathrm{SW}}$ is coupled to the mirror as well (Figure 1), so when moving the mirror along the optical axis *z*, $I_{\mathrm{SW}}$ will also shift, which allows for a length measurement by detecting the local intensity change of the standing wave.

### 2.2. Detection of an Optical Standing Wave

The local intensity of the standing wave in Figure 1 can be detected by a thin transparent photo sensor [7] which is inserted in the optical path at a distance of $z_{s}$ from the mirror position $z_{m}$. This sensor is located directly inside the standing wave and therefore has to be sufficiently transparent and significantly thinner then the optical wavelength. These requirements can not be fulfilled by classical photo detectors. Instead, special Standing Wave Sensors (*SWS*s) are necessary. These *SWS*s can detect the local intensity of the standing wave, so when the *SWS* or the mirror is moved along the optical axis *z*, the periodic profile $I_{\mathrm{SW}}$ of the standing wave will shift through the *SWS*, resulting in an alternating sensor signal (in general a photo current). Counting the minima and maxima of this signal allows for length measurements, when the wavelength λ of the laser source is known.

### 2.3. Standing-Wave Interferometer

**Figure 2:**
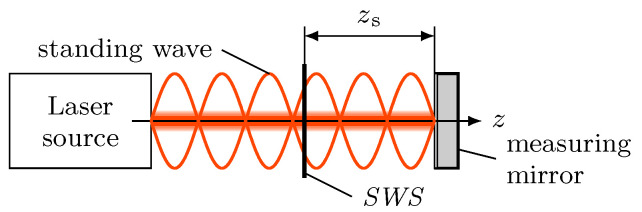
Detection of the standing wave intensity profile by a thin and transparent photodetector (*SWS*). Moving either the *SWS* or the mirror along the optical axis *z* will shift the nodes and antinodes of the standing wave through the sensor, resulting in a pulsating photo signal which can be used for length measurements.

The described principle directly leads to the setup of an *SWI*. This type of interferometer only consists of a laser source, a moving measuring mirror and a thin and transparent *SWS* for detecting the local intensity of the standing wave (Figure 2). This means, the *SWI* has a simple, linear structure with a cross section only slightly larger than the diameter of the laser beam. Furthermore, no beam splitting like in a classic Michelson-interferometer is necessary.

### 2.4. Requirements for the SWS

The requirements for *SWS*s, utilised in a *SWI*, are described in detail in the following sections.

#### 2.4.1. Transparency

The *SWS* is inserted into the beam path of the standing wave and therefore directly affects the electric field. As the incident and reflected wave need to have the same amplitude (Equation (4)), the *SWS* has to be sufficiently transparent to fulfil this requirement. When the amplitude of both waves is different, the intensity of the standing wave will not be zero in the minima, resulting in a direct component of the sensor signal and a reduced amplitude of the alternating component. Hence, the usable amplitude of the sensor signal will be reduced, causing a decrease of the signal-to-noise-ratio, which in general is undesired for length measurement applications. Of course, the sensor transparency is subject to an optimisation between the absorbed energy in the *SWS* (and hence the amplitude of the sensor signal) and the equality of the incident and reflected beam intensity (hence the direct component of the sensor signal). As a trade-off, a transparency of approx. 70% is considered adequate.

#### 2.4.2. Thickness

For the thickness of the *SWS* there are two extremes: with a thickness of zero, obviously there will no sensor signal at all, as no light is absorbed by the *SWS*. The other extreme is a thickness of multiples of $\frac{\lambda }{2}$. In this case, there will be an integration over a whole period of $I_{\mathrm{SW}}$. The consequence then is a constant signal, independent of the relative position between *SWS* and mirror. Both cases are useless for length measurements with an *SWI*. The optimal thickness is located between these two extremes at approx. $\frac{\lambda }{4}$. As the principle of the *SWI* involves optical wavelengths, this means a sensor thickness of only a few nanometres.

#### 2.4.3. Flatness

Because of the geometry of the *SWS*, there is a strict demand for the flatness of the sensor. Every deviation from a perfectly flat geometry leads to decreasing signal contrast. The reason is, different zones of the *SWS* are located at different distances to the measuring mirror and hence detect different local phase angles of $I_{\mathrm{SW}}$. However, in the photo active volume of the *SWS*, all local phase angles will be integrated. This results in a decrease of the amplitude of the alternating sensor signal component, while the direct component will increase. An acceptable flatness of the *SWS* is in the range of $\frac{\lambda }{2}$, which rises high demands for the sensor manufacturing process.

#### 2.4.4. Alignment

A similar problem applies to the orientation of the sensor plane with respect to the optical axis. When the *SWS* is tilted, different zones will again detect different phase angles of $I_{\mathrm{SW}}$ with the described consequences. Fortunately, this requirement can be achieved comparatively easy, as the *SWS* can be adjusted by simple mechanical means in the final *SWI* setup.

#### 2.4.5. Mechanical Stability

Because of the requirements described above, the aspect ratio of the *SWS* is extremely unfavourable: the thickness is in the range of nanometres, while the photoactive area is in the size of the cross-section of the laser beam, so at least 1 mm in diameter. Therefore, the *SWS* represents an ultra-thin membrane, which is mechanically unstable. To guarantee the required flatness of the *SWS*, additional effort needs to be taken. In our project, the *SWS* was bonded to a glass plate for mechanical stabilisation, where the focus lay on a bonding process free from mechanical stress, which in turn would have led to deformations of the *SWS*.

#### 2.4.6. Reflectivity

As a matter of the principle of the *SWI*, *SWS* and measuring mirror have both to be adjusted exactly perpendicularly to the optical axis. This is equivalent to an exact parallel alignment of *SWS* and mirror. Hence, these to components build up an optical cavity, where multiple reflections and multi-beam interferences will arise. These interferences lead to distortions of the sensor signal in the form of harmonics and to periodic non-linearities, which are, in general, undesired for precision length measurements. As the parallel alignment of *SWS* and measuring mirror is essential for the principle of the *SWI*, the only way to suppress those multiple reflections is the reduction of the cavity finesse by reducing reflection coefficient. Here, the measuring mirror can not be modified. From Section 2.1 it can be seen, that incident and reflected wave need to have the same intensity. Otherwise, there will be a direct component in the intensity profile of the standing wave, causing a loss of contrast for the sensor signal. So the remaining possibility is reducing the reflection coefficient of the *SWS*. As the sensors are made of Si (see next passage), they have a high reflective coefficient in air of approx. 50% [2] at first. This high reflectivity can be reduced by adding anti-reflection layers to the basic sensor. To determine the correct thickness of those layers, the matrix method was used [8]. Details of the anti-reflection process can be found in [9]. Summarised, there is the need for a system of anti-reflection layers on both sides of the *SWS* to reduce its reflection coefficient below 1%.

#### 2.4.7. Cut-Off Frequency

A relative movement of the intensity profile $I_{\mathrm{SW}}$ with respect to the photoactive layer of the *SWS* will shift the minima and maxima of $I_{\mathrm{SW}}$ through the sensor, resulting in a periodic sensor signal. The frequency $f_{\mathrm{SWS}}$ of the signal depends on the wavelength λ of the used light source as well as on the relative velocity $v_{z}$ between standing wave and *SWS*. If the time-dependent relative position of *SWS* is inserted into Equation (5), the resulting sensor signal will have a frequency of

(6) $$f_{\mathrm{SWS}}=2\frac{v_{z}}{\lambda }\;.$$

To be able to track fast movements of the *SWS* respectively the mirror, the *SWS* must have the ability to detect the intensity profile $I_{\mathrm{SW}}$ with a sufficient speed. Otherwise, if the frequency is to high, pulses will be lost resulting in a length measurement error. As an estimation, a maximum velocity of $v_{z,\mathrm{SWS}}=1\;m\;s^{-1}$ is considered to be sufficient for most length measuring tasks in the aspired area of application. For a typical He-Ne laser with λ=633 nm, this results in a frequency of $f_{\mathrm{SWS}}=3.16\mathrm{MHz}$ (Equation (6)). The cut-off frequency of the *SWS* has therefore to be higher than this value, so the *SWI* can be used for various applications without any restrictions.

### 2.5. Direction Discrimination

Setting up a *SWI* with a single photo detector (Figure 2) will suffer from the missing possibility for a reliable determination of the moving direction of the measuring mirror respectively the *SWS* at every time. Therefore, for the homodyne *SWI* two transparent photo sensors are required for detecting the local intensity of $I_{\mathrm{SW}}$ at different positions along the optical axis for obtaining two phase shifted signals. For utilising a state-of-the-art arctan-demodulation [10], two exactly 90° phase shifted quadrature signals are required, hence the distance between the two sensors has to be adjusted to $\left(2n-1\right)\frac{\lambda }{8}$.

However, the second *SWS* introduces several problems for the homodyne *SWI*. First, the additional optical material in the beam path leads to additional wavefront distortions, reflections and absorption. This again lowers the signal-to-noise-ratio, as described in the sections before. The main weak point, however, is the sensor-to-sensor distance. This parameter is crucial for obtaining quadrature signals with the correct phase shift of 90° and has to be exactly manufactured or adjusted with nanometre accuracy. Furthermore, this distance has to be thermal and long-term stable, otherwise a phase shift between the two sensor signals will not equal 90°, leading to periodic non-linearities in the length measurement. To overcome this strict requirements, a different approach for obtaining quadrature signals with only a single *SWS* is proposed in this paper. In this approach, the measuring mirror is oscillated and the resulting harmonics in the sensor signal are used for phase demodulation.

## 3. Experimental Setup and Results

Oscillating one of the mirrors in a Michelson-interferometer is in general done for the purpose of obtaining alternating interference signals to compensate drift effects in offset or amplitude. This way, drifts in the length signal and periodic non-linearities of the interferometer can be significantly reduced, as shown in [11]. In contrast, in a *SWI*, the measuring mirror is modulated for obtaining quadrature signals for direction discrimination.

### 3.1. Quadrature Signal Generation

To obtain quadrature signals for direction discrimination from a single *SWS*, the approach in this project uses a phase modulated sensor signal. This is achieved by superimposing the movement of the measuring mirror by a forced oscillation with the amplitude $z_{\mathrm{ca}}$ and a carrier frequency $f_{\mathrm{ca}}$. The basic principle of this approach has already been demonstrated for a Michelson-interferometer [12]. In the described *SWI*, the sensor signal $s_{\mathrm{SWS}}$ can be calculated by

(7) $$s_{\mathrm{SWS}}=\cos\left(\frac{4\pi }{\lambda }\left(z_{s}+z_{\mathrm{ca}}\sin\left(2\pi f_{\mathrm{ca}}t\right)\right)\right)\;.$$

**Figure 3:**
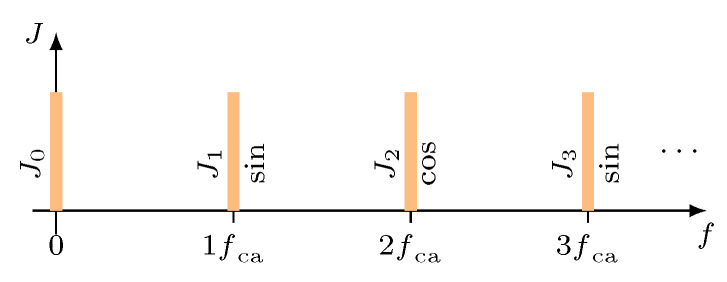
Resulting spectrum of the sensor signal $s_{\mathrm{SWS}}$ with a modulated measuring mirror and a carrier frequency $f_{\mathrm{ca}}$ (amplitudes not to scale). The actual amplitude $J_{n}$ of each spectral component depends on the wavelength λ of the laser source and the modulations stroke $z_{\mathrm{ca}}$ [13].

The phase-modulated signal $s_{\mathrm{SWS}}$ contains equidistant spectral components (harmonics) with a frequency distance of $f_{\mathrm{ca}}$, which are alternating coupled with the sine (odd multiples of $f_{\mathrm{ca}}$) or with the cosine (even multiples of $f_{\mathrm{ca}}$) of the sensor position $z_{s}$. Figure 3 shows a part of the resulting spectrum.

To obtain quadrature signals from the modulated sensor signal, it is necessary to extract at least two spectral components: one sine- and one cosine-component. In the described *SWI*, this is achieved by using a lock-in technique. There, the sensor signal $s_{\mathrm{SWS}}$ (Equation (7)) is multiplied with the signal $s_{\mathrm{LO}}$ of a Local Oscillator (LO) with a frequency $\left(f_{\mathrm{LO}}\right)=\left(2n-1\right)f_{\mathrm{ca}}$ to generate the sine quadrature signal, respectively a second LO with $f_{\mathrm{LO}}=2nf_{\mathrm{ca}}$ for the cosine quadrature signal. As both quadrature signals are located in the base band after mixing and filtering, it is possible to use the standard arctan-demodulation procedure afterwards for determination of the sensor signal phase. In general, all spectral components are suitable for this approach. As an example, the sine-component can be obtained from the first spectral component at $f_{\mathrm{ca}}$:

(8) $$\begin{array}{cc} s_{\mathrm{SWS}}\;\cdot \;{s_{\mathrm{LO}}}_{1} & =J_{1}\left(\frac{4\pi }{\lambda }z_{\mathrm{ca}}\right)\sin\left(\frac{4\pi }{\lambda }z_{s}\right) \end{array}$$

(9) $$\begin{array}{cc} \; & =s_{\sin}\sin\left(\frac{4\pi }{\lambda }z_{s}\right)\;. \end{array}$$

The cosine-component can be obtained from the second spectral component at $2f_{\mathrm{ca}}$:

(10) $$\begin{array}{cc} s_{\mathrm{SWS}}\;\cdot \;{s_{\mathrm{LO}}}_{2} & =J_{2}\left(\frac{4\pi }{\lambda }z_{\mathrm{ca}}\right)\cos\left(\frac{4\pi }{\lambda }z_{s}\right) \end{array}$$

(11) $$\begin{array}{cc} \; & =s_{\cos}\cos\left(\frac{4\pi }{\lambda }z_{s}\right)\;. \end{array}$$

**Figure 4:**
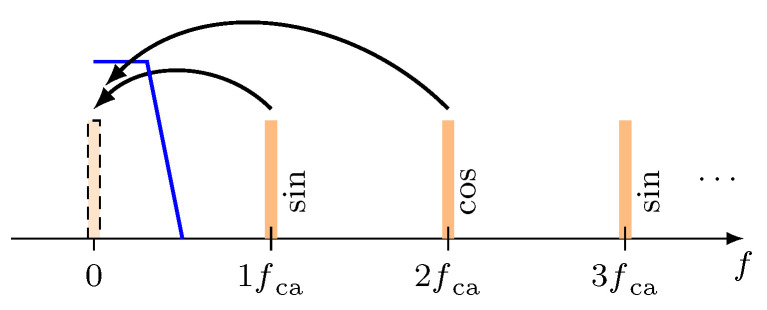
Shifting of the first ($1f_{\mathrm{ca}}$) and second ($2f_{\mathrm{ca}}$) spectral component to the base band by mixing and low-pass filtering (blue line).

The described steps represent a shift of the first and second spectral components to the base band (Figure 4) where they can be evaluated by means of a state-of-the-art arctan-demodulation.

**Figure 5:**
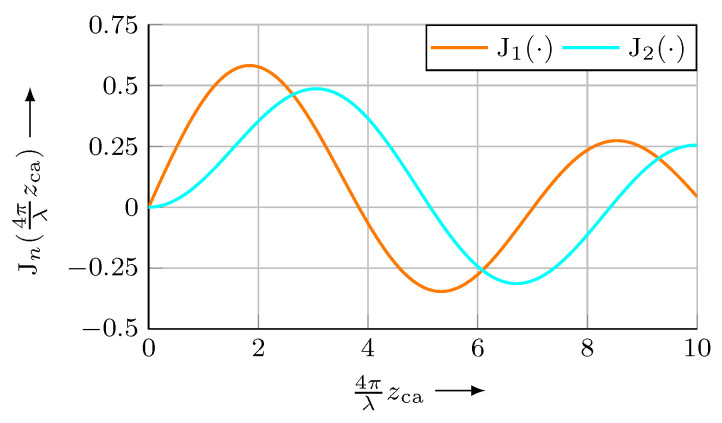
Amplitudes $J_{n}$ of the first two spectral components, depending on the modulation stroke $\frac{4\pi }{\lambda }z_{\mathrm{ca}}$.

Using two of the first few spectral components has the advantage of a higher signal amplitude compared to higher order harmonics. Furthermore, lower frequencies are, in general, easier to handle from an electronic point of view. As the amplitudes $s_{\sin}$ and $s_{\cos}$ of the obtained signals depend on the ratio of the modulation stroke $z_{\mathrm{ca}}$ and the wavelength λ (Figure 5), $z_{\mathrm{ca}}$ has to be carefully adjusted.

For the arctan-demodulation, it is necessary that $s_{\sin}=s_{\cos}$. Otherwise, periodic non-linearities will arise, which have to be laboriously compensated. The equality of both amplitudes could in theory be achieved by selecting an arbitrary or free floating $z_{\mathrm{ca}}$ and a variable gain amplifier for each quadrature component, continuously adapted to the current obtained amplitude. However, the risk of accidentally approaching a $z_{\mathrm{ca}}$, where any of the utilised $J_{n}=0$ (see Figure 5), e.g., due to (thermal) drift effects, exists in this case. Thus, as $z_{\mathrm{ca}}$ has to be controlled anyway, a convenient solution is monitoring the current amplitudes $s_{\sin}$ and $s_{\cos}$, and tuning the modulation stroke $z_{\mathrm{ca}}$ accordingly in a closed loop control. From Equations (8) and (10) it can be deduced, that for this purpose the condition

(12) $$J_{1}\left(\frac{4\pi }{\lambda }z_{\mathrm{ca}}\right)=J_{2}\left(\frac{4\pi }{\lambda }z_{\mathrm{ca}}\right)$$

has to be fulfilled. This can be achieved at every point of intersection of $J_{1}$ and $J_{2}$ (see Figure 5). Once again, for a maximum signal amplitude, the first intersection at $\frac{4\pi }{\lambda }z_{\mathrm{ca}}\approx 2.63$ is preferred. When generating the modulation signal, the phase shift along the processing path electronics–modulation device–*SWS*–electronics has to be considered. Any phase shift between the LO and the sensor signal $s_{\mathrm{SWS}}$ will lead to amplitude deviations of the sin- and cosine-component, leading to periodic non-linearities, as decribed above.

**Figure 6:**
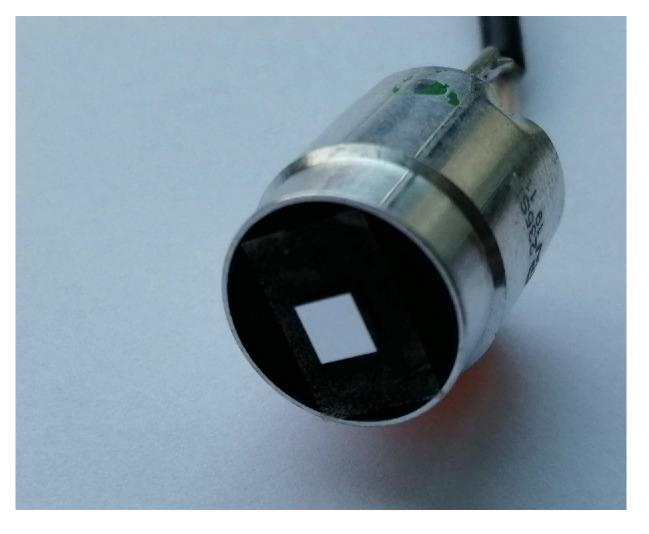
UltraSonic Transducer (*UST*) with measuring mirror for the *SWI*. With this setup, the mirror position can be modulated with a frequency $f_{\mathrm{ca}}$, additionally to the underlying measurement motion.

The phase modulation between *SWS* and measuring mirror can be achieved by different means. Here, the preferred methods are modulating the position of the *SWS* or the position of the measuring mirror. Though modulating the laser wavelength allows $f_{\mathrm{ca}}$ in the MHz range when using laser diodes [14], this attempt is not suitable for a He-Ne laser. Furthermore, the modulation stroke $z_{\mathrm{ca}}$ has to be continuously adapted to the absolute distance between *SWS* and mirror. Therefore, the modulation was realised by a forced oscillation of the measuring mirror. For that purpose, a small mirror (3 mm × 3 mm) was glued onto the emitting surface of a commercial UltraSonic Transducer (*UST*) (Figure 6). This way, the movement of the mirror, induced by the measuring motion can be superimposed by an oscillation with the correct stroke $z_{\mathrm{ca}}$ and a frequency $f_{\mathrm{ca}}$. For a He-Ne laser with λ=633nm, the required modulation stroke is 132 nm (Equation (12), Figure 5).

### 3.2. Sensor Technology

As explained in Section 2.4, special transparent photo sensors are required for the operation of a *SWI*. The *SWS*s in this project were developed based on standard semiconductor technologies and materials to enable an economic mass production. The state of the art and the manufacturing of the *SWS*s is extensively described in [9], the following passage gives a brief summary of the process.

**Figure 7:**
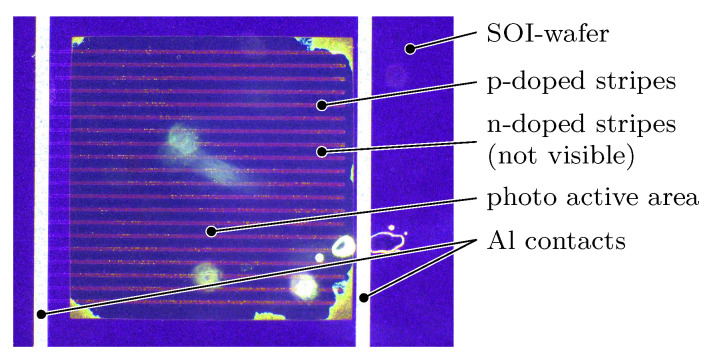
Front view of the standing-wave sensor. The transparent photo-active area has a size of approx. 1 mm × 1 mm. The p- and n-doped stripes are connected with aluminium conductors for further connection [16].

The base material for the *SWS*s is a Silicon On Insulator (*SOI*) wafer, which enables the described sensor requirements. The photo active element is realised as a common p-i-n photo diode. However, the p-i-n pattern defines the thickness of the *SWS*. As explicated in Section 2.4.2, the optimal value is λ/4 which results in a thickness of only 41 nm for a photo diode made of silicon with a refractive index of 3.87 [15] and a laser wavelength of 633 nm. When using the conventional structure of a p-i-n diode with the p, i and n layers arranged consecutively stacked along the optical axis, the intermediate i layer defines the optical relevant thickness of the *SWS* and thus has to be close to 41 nm. However, the resulting extremely tight distance between the p and n layer in combination with the size of the cross section of the *SWS* leads to a comparatively high junction capacitance and hence a low cut-off frequency $f_{\mathrm{co}}$. Therefore, a striped p-i-n profile is doped in the thin upper silicon layer of the *SOI* wafer by ion implantation, forming a lateral p-i-n photo diode (Figure 7) with a thickness of only a few nanometres. This way, the distance between the p and n layer defining the junction capacity can be designed independently of the thickness of the photoactive layer, allowing for excellent dynamic and electric properties of the *SWS*.

**Figure 8:**
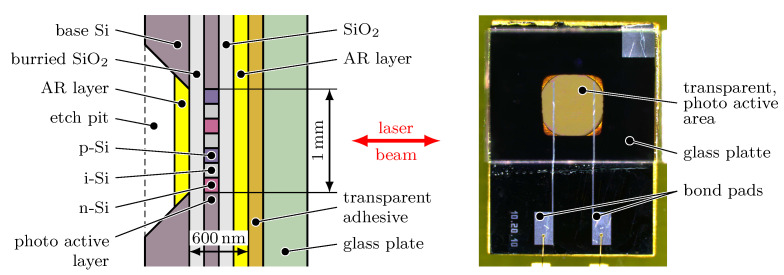
Left: Cross section of the standing-wave sensor. The photo active layer is electrically isolated by surrounding SiO${}_{2}$-layers. For transparency, the bulk silicon is etched in the region of the p-i-n diode. Additionally, the front and rear side of the sensor is anti-reflection (AR) coated. The glass plate stabilises the ultra-thin membrane (thickness ≈600nm). Right: Detail view of the SWS with transparent photo active area and stabilising glass plate. View along the optical axis [16,17].

Up to now, the processed photo diodes can not yet serve as a *SWS*, as the bulk silicon with a thickness of approx. 0.3 mm is still in place and is non-transparent in the visible wavelength range. For that reason, the bulk silicon on the rear side of the wafer has to be locally removed in the area of the p-i-n photo diode to achieve transparent standing-wave sensor. However, when removing the bulk silicon without additional precaution, the *SWS* forms an ultra-thin membrane with a thickness on only 600 nm (including the actual photo active layer and surrounding layers) and a cross section of approx. 1mm×1mm. This membrane is mechanically extremely delicate and will deform immediately due to the inherent mechanical stress in the different sensor layers. Hence, the *SWS* is stabilised by a glass plate prior to the etch process for the bulk silicon to fulfil the demands for the flatness of the photo active layer (Section 2.4.3 and Section 2.4.5). Finally, two anti-reflection layers are deposited on the front and the rear side of the wafer to reduce the reflection coefficient of the whole layer system (Section 2.4.6, Figure 8).

The *SOI* technology enables the fabrication of the *SWS* with standard semiconductor materials and processes capable for series production. Compared to conventional interferometers, the manufacturing costs can be significantly lowered, opening up completely new fields of application for interferometric measurement methods.

### 3.3. Basic Setup of the Phase Modulated Standing-Wave Interferometer

**Figure 9:**
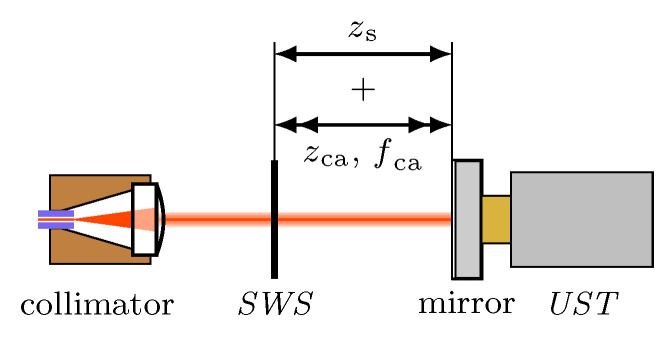
Setup of the phase modulated *SWI*. The motion of the measuring mirror along the optical axis *z* is superimposed by a forced oscillation with a frequency $f_{\mathrm{ca}}$, induced by an UltraSonic Transducer (*UST*).

The basic experimental setup is derived from the basic layout in Figure 2. Additionally, the *UST* for modulating the measuring mirror Section 3.1) is integrated in the system. The whole interferometer is as simple as shown in Figure 9 and only consists of a fibre coupled He-Ne laser, a modulated measuring mirror and the single transparent standing wave sensor.

### 3.4. Signal Processing

The processing of the sensor signal acquired in a *SWI* according to Figure 2 involves several steps to implement the theoretical process described in Section 3.1. To flexibly establish a signal processing hardware, a test and measurement board based on a system-on-chip was used. The system contains all required hardware: Digital-to-Analog Converters (*DAC*s) for generating a modulation signal for the *UST*, Analog-to-Digital Converters (*ADC*s) for sampling the sensor signal $s_{\mathrm{SWS}}$, a Field Programmable Gate Array (FPGA) for real-time computations (mixing, filtering, arctan-demodulation) and a Central Processing Unit *CPU* for running an operating system for user interaction.

**Figure 10:**
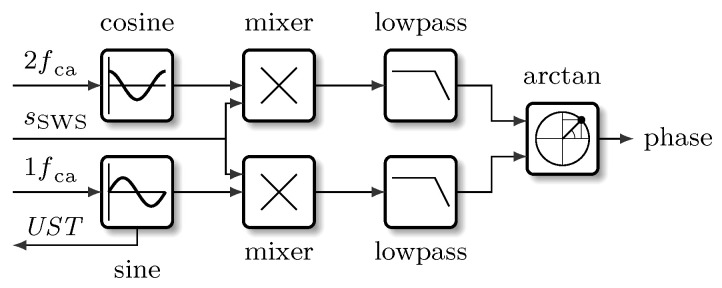
Signal processing of the phase modulated *SWI* for determining the phase of the sensor signal $s_{\mathrm{SWS}}$ by applying a lock-in technique. By mixing and filtering with the appropriate frequencies, the spectral components of $s_{\mathrm{SWS}}$ are shifted into the base band. Then, a conventional arctan-demodulation is utilised for determining the phase angle of $s_{\mathrm{SWS}}$.

The algorithm for transferring the first and second spectral component to the base band by mixing and filtering is depicted in Figure 10 and was implemented in the *FPGA* part of the system-on-chip.

**Figure 11:**
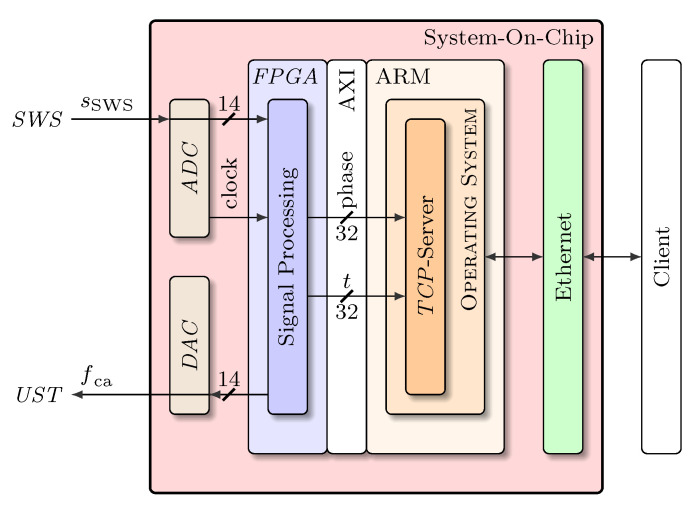
Scheme of the signal processing and communication. The signal $s_{\mathrm{SWS}}$ from the *SWS* is digitised and the phase angle is calculated by the *FPGA* (Figure 10). The phase value and a timestamp *t* are transferred via an Advanced Extensible Interface (AXI) to the operating system and from there distributed to the client(s) via Transmission Control Protocol (*TCP*).

On the test and measurement board, the signal of the *SWS* is analogue/digital converted and then processed by the algorithm shown in Figure 10 in the *FPGA* part of the system. The calculated phase angle as well as a timestamp are transferred via an Advanced Extensible Interface (AXI) from the *FPGA* to the operating system and made available via an Ethernet connection to the client computer(s), see Figure 11. Further information about the system and the algorithm can be found in [9].

### 3.5. Theoretical Limits of the Proposed *SWI*

As in every realisation of an interferometric measuring technique, there are limitations regarding resolution, accuracy and measuring distance for the phase modulated *SWI*.

The interference of incident and reflected wave as well as the mirror modulation are pure analogue processes, hence the resolution of the phase modulated *SWI* is essentially limited by the resolution of the signal processing chain. In the current setup, the limiting component is the utilised 14 bit *ADC*, all subsequent calculation processes in the *FPGA* are carried out with at least 16 bit and could even use broader resolution. The smallest detectable moving step Δz of the measuring mirror in an interferometer can be calculated by

(13) $$\Delta z=\frac{\lambda }{i\;2^{N_{b}}},$$

where *i* is the interferometer factor and $N_{b}$ is the number of bits. As the phase modulated *SWI* is a λ/2 interferometer, i=2 is applied. Thus, for a wavelength of λ=633nm and an *ADC* with $N_{b}=14$, the smallest detectable moving step is Δz=19pm. Resolution can be increased by oversampling and averaging [18]. This way, the dynamic range for the phase measurement of $s_{\mathrm{SWS}}$ can be increased over the dynamic range of the utilised *ADC*, by the cost of dynamics.

The overall accuracy of the phase modulated *SWI* depends on many aspects like stability of the laser frequency, refractive index *n* in the optical path $z_{s}$ (air and parts of the *SWS* itself), thermal drift effects and periodic non-linearities. Quantifying these factors of influence and estimating the resulting measuring uncertainty in theory is described in detail in [9].

The minimal and maximal measuring distance of the phase modulated *SWI* strongly depends on the source of the modulation. Modulating the laser wavelength results in the necessity to tune the modulation stroke according to the current distance $z_{s}$ between *SWS* and measuring mirror (Section 3.1) [14]. There, the limitation of the measuring length arises from the range, the modulation stroke can be adjusted. As the required modulation amplitude is inverse to the measuring distance, the minimal length is limited by the maximal wavelength stroke and vice versa, the maximum distance is limited by the minimal possible stroke. Furthermore, when modulating the laser wavelength, there is a fundamental limitation for the measuring range of $z_{s}<c\;/\;2f_{\mathrm{ca}}$, which cannot be enlarged by any means [19].

However, for the proposed configuration of modulating the sensor–mirror distance (Figure 9) instead of the laser wavelength, this restrictions do not apply. There, the modulation phase is direct proportional to the mechanical modulation stroke of the mirror respectively the *SWS* (Figure 5 and related equations), independent of the current distance $z_{s}$.

Thus, the maximum measuring distance is only limited by the coherence length *L* of the laser source, which is $L\approx 0.44\;\lambda^{2}\;/\;n\Delta \lambda$, with Δλ, the full-width-half-maximum spectral width of the laser source [20], which is in the range of 100 m for common He-Ne lasers [21].

### 3.6. Measurements

**Figure 12:**
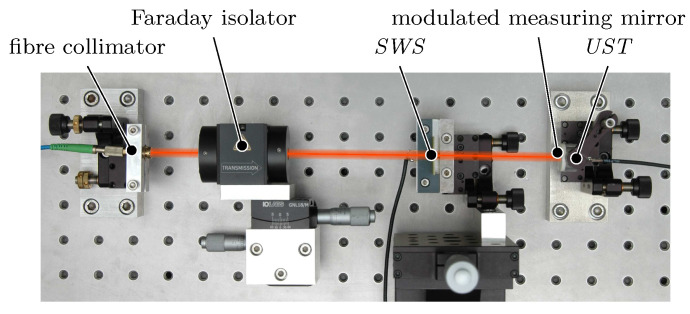
Verification setup for the phase modulated *SWI*. Additionally to Figure 9, a Faraday isolator was added to suppress reflections from the measuring mirror back into the laser source. The *UST* is fixed, resulting in a modulation of the sensor position around a static position *z* with a frequency $f_{\mathrm{ca}}$.

Before setting up an interferometer for length measurements, the basic principle of the phase modulated *SWI* was validated in a simplified verification setup. There, the detection of the standing wave intensity profile by the *SWS* (Section 2.1), the generation of harmonics in the sensor signal by the *UST* (Section 3.1) and the amplitudes and spectral distances of the signal components were investigated. In the verification setup, the *SWS* was mounted on a fixed *UST*, so the sensor signal was only affected by the modulation amplitude $z_{\mathrm{ca}}$ and the carrier frequency $f_{\mathrm{ca}}$. Figure 12 shows the layout of the verification setup.

**Figure 13:**
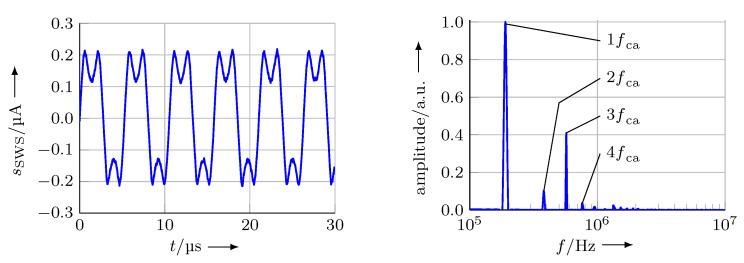
Left: signal of the fixed *SWS* in a setup according to Figure 12. Right: The Fourier transform of the signal reveals the spectral components at the resonance frequency of the *UST* $f_{\mathrm{ca}}=190\mathrm{kHz}$ and multiples thereof at $2f_{\mathrm{ca}}$, $3f_{\mathrm{ca}}$ and $4f_{\mathrm{ca}}$. The amplitudes of the spectral components depend on the modulation stroke (Equations (8) and (10) correspondingly apply also for the higher order harmonics).

In this setup, the signal $s_{\mathrm{SWS}}$ of the fixed *SWS* was recorded. From Section 3.1 it can be deduced, that in this situation the sensor signal should contain several spectral components of different amplitudes, depending on the modulation stroke $z_{\mathrm{ca}}$ of the measuring mirror (Equations (8) and (10)), equally spaced with a spectral distance of $f_{\mathrm{ca}}$. Figure 13 shows the raw sensor signal as well as its normalised spectrum.

After the basic investigations according the sensor signal and spectral components were done, interferometric measurements with the described principle were carried out. For that purpose, the *SWS* respectively the modulated measuring mirror were mounted on different linear axes to produce relative distance modifications between *SWS* and mirror. These motions induce a shift of the modulated intensity profile of the standing wave, which was detected by the *SWS*, demodulated and evaluated.

**Figure 14:**
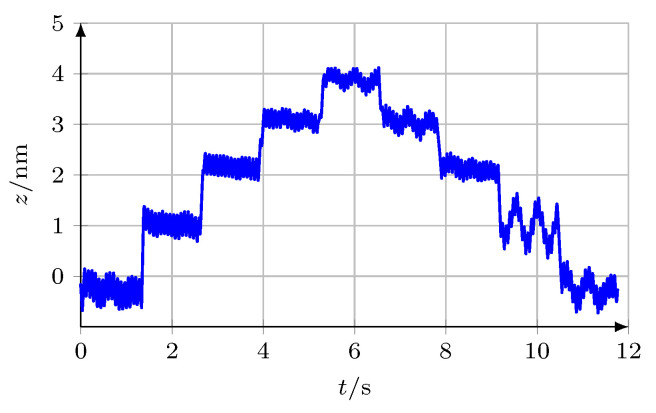
Staggered motion of the oscillating measuring mirror along the optical axis, average step height is 1.1 nm.

A major reason for using interferometric length measurement systems is their high resolution in the nanometre range. As a proof of the resolution capability of the *SWI*, the *UST* carrying the measuring mirror was mounted on an additional piezo actuator (cascaded driving system) where the voltage applied was increased and decreased stepwise. This results in a staggered motion of the mirror (nanometre steps back and forth along the optical axis), superimposed by the forced oscillation. The calculated stepwise position of the mirror is depicted in Figure 14, the mean step height is 1.1 nm.

**Figure 15:**
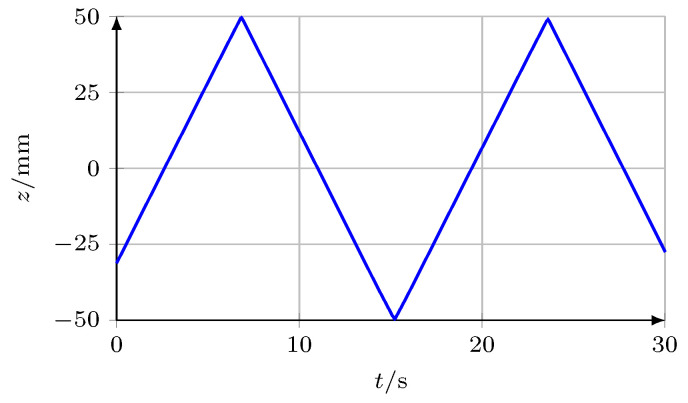
Alternating movement of the *SWS* over 100 mm. The maximum velocity between the reversal points was 12 $\mathrm{mm}\;s^{-1}$.

To prove the ability of the *SWI* for high measuring velocities over large moving distances, the *SWS* was mounted on a motorised linear axis, while the *UST* was at a fixed position. Then, the *SWS* was moved with a velocity of 12 $\mathrm{mm}\;s^{-1}$ over the complete positioning range of 100 mm of the axis. The signal $s_{\mathrm{SWS}}$ of the *SWS* was sampled by the test and measurement board (Section 3.4) and the described signal processing algorithm was executed. The demodulated length information is depicted in Figure 15.

Figure 15 shows the ability of the phase modulated *SWI* to track motions with high velocity over large distances without any data loss or miscounting of interference fringes. Thus, the developed *SWI* is capable of high resolution measurements in large measuring ranges and with comparably high measuring velocity and thus should be suitable for most measurement tasks in industry.

## 4. Conclusions and Outlook

In this manuscript, a new interferometer principle is proposed, which is based on detecting the intensity profile of an optical standing wave with an ultra-thin, transparent photo sensor. The standing wave arises, when a laser beam is perpendicularly reflected at a mirror, and then incident and reflected beam interfere in opposite directions. The resulting intensity profile consists of equally spaced minima and maxima, which are coupled to the mirror surface. So when the mirror is moved along the optical axis, the periodic intensity will also shift, enabling an interferometric length measurement, when the minima and maxima are counted by detecting the local intensity of the standing wave at a fixed position. This detection can be realised by an ultra-thin and transparent photo detector. This standing wave sensor is located in the optical beam path of the standing wave and therefore has to meet several requirements including transparency, thickness, flatness and reflection coefficient.

As the position signal of the interferometer is phase modulated, quadrature signals for determining the moving direction of the measuring mirror can be derived from a single intensity signal. Thus, in the proposed setup, there is no need for using two standing wave sensors, which has shown to be very challenging with respect to the sensor-to-sensor distance and the optical interaction between the two sensors.

The sensors for detecting the standing wave intensity profile have been successfully fabricated, fulfilling the appropriate requirements for a *SWI*. The production technology is based on standard semiconductor materials and processes, enabling a cheap mass production of interferometric systems. This suits the simple and extremely compact design of the standing wave interferometer, opening up new fields of applications for interferometric measurements.

The ability for detection of the standing wave was proved for a sensor at rest with the measuring mirror oscillated with an *UST*. The capability of detecting small positioning steps of the measuring mirror as well as tracking fast movements over large ranges have also been demonstrated in this manuscript.

The demonstrated parameters of the phase modulated *SWI*, in combination with the very simple, compact and linear setup makes this type of interferometer suitable for applications in industry and engineering, where installation space is an issue. Furthermore, the possibility for a cheap mass production of the *SWS* opens up the chance for fields of application, where interferometric measurements have been out of question so far. This includes linear stages, machine tools and length measuring systems in quality control or production measurement technology (measuring probes etc.).

In future works, the oscillation frequency of the measuring mirror has to be increased further, as the moving velocity of the *SWS* respectively the measuring mirror is limited by this parameter. Currently, the modulation frequency of the *UST* is 190 kHz, resulting in a maximum velocity of 30 $\mathrm{mm}\;s^{-1}$.

A useful improvement will incorporate the modulation of the *SWS* instead of the measuring mirror. This way, the *UST* is placed at the standing wave sensor which requires an electrical connection anyway. Also an integration of the modulation device in the *SWS* itself is possible. With the modulation realised in such way, the measuring mirror is completely passive and unleashed from the active part of the interferometer.

Finally, a user-friendly control and evaluation platform has to be developed. Up to now, the modulation stroke and phase are tuned by hand by the operator and have to be frequently readjusted due to to thermal drifts of the *UST*. To reduce the effort during installation and operation, this tuning will be executed by the control electronics in the future. This can be realised comparatively easy by monitoring phase and amplitude of two spectral components (Figure 5). 

## Data Availability

Data available on request.
